# IL-8 mediates a positive loop connecting increased neutrophil extracellular traps (NETs) and colorectal cancer liver metastasis

**DOI:** 10.7150/jca.44215

**Published:** 2020-05-18

**Authors:** Luyu Yang, Lu Liu, Rui Zhang, Jun Hong, Yaping Wang, Jian Wang, Jieliang Zuo, Jubo Zhang, Jinhong Chen, Hankun Hao

**Affiliations:** 1 Department of General Surgery, Huashan Hospital, Fudan University, Shanghai, China; 2 Caner Metastasis Institute, Fudan University, Shanghai, China; 3 Department of Infection Disease, Huashan Hospital, Fudan University, Shanghai, China

**Keywords:** colorectal cancer (CRC), liver metastasis, Neutrophil extracellular traps (NETs), IL-8

## Abstract

Host and tumorous inflammation actively affect liver metastasis of colorectal cancer (CRC). Neutrophils have been recognized as one active participant in metastasis procedure, with controversial roles however. Activated neutrophils release extracellular traps (NETs) which are involved in infection and multiple pathological conditions. NETs on cancer metastasis is getting recognized but less elucidated in mechanism. How NETs interact with cancer cells is still largely unknown. In this study, we found that neutrophils from CRC patients, especially those with liver metastatic, underwent remarkably enhanced NETs. Clinically, sera and pathological NETs marker closely correlated with onset of liver metastasis. Through *in vivo* and *in vitro* studies, we proved that increased NETs positively contribute to onset of CRC liver metastasis. Digesting NETs with DNase 1 diminished the increased liver metastasis associated with NETs. In detail, NETs trapped CRC cells in liver and exerted no cytotoxicity on tumor cells, but boosted tumorous proliferation and invasion capacity. We further found this enhanced malignancy of trapped CRC cells was due to the elevated tumorous interleukin (IL)-8 expression triggered by NETs. Blocking IL-8 activity effectively abrogated the enhanced proliferation and invasion triggered by NETs. Moreover, overproduced IL-8 in turn activate neutrophils towards NETs formation, thus forming a positive loop optimizing CRC liver metastasis. Collectively, our study propose a novel positive feedback between elevated tumorous IL-8 and NETs to promote CRC liver metastasis, and identify potential strategy against liver metastasis.

## Introduction

Colorectal cancer (CRC) is the third most common cancer and the fourth most common cancer cause of death globally. Half of CRC patients eventually develop liver metastasis 1. The multiple interactions among cancer cells and host immune cells in liver complicate the formation of liver metastasis 2. The mechanism of how CRC liver metastasis is developed is still not fully understood. Cancer is closely related to inflammation and meanwhile inflammation complicates cancer metastasis 3. Neutrophils are the most abundant host leukocytes and play vital role in inflammation. The rapid mobilization and recruitment of neutrophils form host first defense line in inflammatory response and help wound healing 4. On the other side, the neutrophils are often hijacked by cancer to favor metastasis 5,6. While some studies claimed neutrophils inhibit metastasis, however 7. An elevation of neutrophils-lymphocytes ratio (NLR) and several inflammatory mediators have been reported in CRC patients correlated with favourable/unfavourable prognosis 8,9. However, the underlying mechanism still remain to be elucidated 10.

Neutrophil extracellular traps (NETs) are large extracellular web-like structures comprised of decondensed chromatin and protease that are released by activated neutrophils under certain stimulus 11-14. NETs have been discovered as an essential role of neutrophils in host defense and immunity regulation 15,16. In several pathological circumstance, neutrophils are activated to form NETs, which are proven essential in infection, autoimmune disease, diabetes and other inflammation-related disorders 17. The presence of NETs are also described in cancer patients and mice model 18-22. Some studies have suggested a pro-tumor role of NETs in cancer metastasis. The trapping role of NETs to optimize the seeding of cancer cells like trapping pathogen in pre-metastatic sites may be one explanation of how NETs are related with metastasis. Despite these, the underlining mechanism is poorly understood. One report has suggested NETs can awaken dormant breast cancer cells in mice 23. Despite these, how NETs participate in CRC liver metastasis and how NET formation is regulated in metastatic CRC are still less studied.

Interleukin-8 (IL-8), also known as CXCL8, is one vital chemokine to attract and activate neutrophils 24. Besides, IL-8 is also related with cancer cell survival, proliferation and invasion, as well as angiogenesis 25,26. IL-8 in the tumor microenvironment, and tumorous over-expression of IL-8 have been implicated in promoting tumor progression in an autocrine or pancrine model 27. Elevated IL-8 has been implied with adverse outcome in a variety of tumor types, including ovarian cancer, breast cancer, lung cancer, gastric cancer, and CRC 25. In this study, we went to access the cross linked role of neutrophil NETs, over-produced IL-8 and CRC metastasis in clinical patients samples as well as cell and mice models. This study may deepen the understanding of how inflammation regulates CRC liver metastasis, and also provide novel therapeutic potentials.

## Material and Methods

### Human specimens, animal models and cell lines

For pathological analysis, formalin fixed and paraffin embedded tissue samples of 16 primary CRC tumors with paired normal colon, 10 primary liver tumors (hepatocellular carcinoma) and another 10 pairs of primary CRC tumor and matched liver metastasis (LMT) were obtained during surgical resection of primary tumor or combined colectomy and hepatectomy in our institute during 2019. For sera analysis or neutrophils isolation, Peripheral blood samples were obtained from 41 CRC patients and 32 healthy donors for sera analysis. All enrolled CRC patients were diagnosed by pathologists following surgical resection or endoscopy and received no previous anti-tumor treatment. The clinical parameters were listed in **Table S1-4**. All samples were obtained under the regulation of the Ethics Committee of Huashan Hospital, Fudan University in agreement with the Declaration of Helsinki with written consent.

Six- to 8-weeks old of C57BL/6 male mice or null-mice were used in animal studies. The human cell line HT29 and mice cell line MC38 were obtained from Chinese Academy of Sciences. All animal experiments were approved by the Animal Ethics Committee of Fudan University.

### NETs formation assay

To evaluate NETs formation capacity, freshly isolated human or mice neutrophils were adjusted to a concentration of 5×10^5^ cells/ml and left untreated or stimulated with following stimulus for indicated hours with or without DNase 1(100U/mL) to allow NETs formation: 20nM PMA (Phorbol 12-myristate 13-acetate, Sigma-Aldrich), IL-8 (10ng/ml, R&D), Lipopolysaccharide (LPS, 50ng/ml, Sigma). In plasma-induced NETs formation assay, neutrophils were incubated with corresponding plasma (1:2) for 3 hours.

For visualization, neutrophils were seeded on 96-well plates for corresponding incubation, and cell-impermeable DNA dye Sytox (Thermo Fisher Scientific, 1:10000) and cell-permeable DNA dye Hoechst 33342 (Thermo Fisher Scientific, 1:1000) were added to the incubation system. At the end of incubation, the plates were directly moved to fluorescence microscope (Leica) for NETs formation visualization. In some cases, neutrophils were seeded on coverslips in 24-well plates to generate NETs as described above, and then the formed NETs were fixed for further immunofluorescence detection.

For quantification, NETs-DNA generated by neutrophils were digested with 500 mU/ml micrococcal nuclease (MNase). The nuclease activity was stopped with Ethylenediaminetetraacetic acid (EDTA, 5 mM) and the culture supernatants were collected and stored at -80°C until further use. NETs-DNA in the supernatants was quantified by PicoGreen® dsDNA Quantitation Reagent (Thermo Fisher Scientific) with fluorescence spectrometry under filter setting of 480 nm/520 nm excitation/emission and semi-quantitatively standardized to control group.

### Preparation of NETs

Neutrophils were isolated and seeded on 6-well plates (1×10^7^/well). Human neutrophils were stimulated with 20nM PMA for 4 hours. Then the supernatants were discharged carefully by slow suction and washed twice to eliminate residual PMA or NETs-unassociated substances without disturbing NETs. RPMI (1ml) containing MNase (1U/ml) was then added to digest NETs at 37°C for 20 minutes followed by 5mM EDTA to stop nuclease activity. The supernatant containing NETs were collected and centrifuged to eliminate cell debris. Isolated NETs were stored at -80°C for further use.

### Measurement of MPO-DNA and IL-8 level

We measured MPO-DNA complexes in human sera using a well-adopted capture ELISA assay with some modification. Briefly, as the capturing antibody, 5µg/ml anti-MPO monoclonal antibody was coated to 96-well plates overnight at 4°C. After blocking in 1% BSA, 100µl of diluted serum was added per well and incubated at room temperature on a shaking device for 2 hours. After washing five times with PBST, PicoGreen® dsDNA Quantitation Reagent was added according to manufacturer's directions. The values were then read with a fluoremeter with a filter setting of 480nm/520nm excitation/emission and semi-quantitatively standardized to healthy donor or control group. IL-8 level was measured following a commercially purchased ELISA Kit used according to the manufacturer's instructions (BD Bioscience).

### Mice model 1: LPS-induced NETs model

LPS (Lipopolysaccharide, 10ug/mouse, Sigma) was given intraperitoneally to induce systemic inflammation in C57BL/6 mice. DNase 1 (100U/mouse) was given intraperitoneally daily as abrogation 24 hours prior to LPS. To verify NETs formation in the inflammation model, mice were sacrificed in 6 hours after LPS injection, livers were removed and embedded in O.C.T. Compound for frozen sections and subsequent *in situ* immunofluorescence staining of NETs.

### Mice model 2: Establishment of experimental metastasis in LPS-induced NETs model

Six hours after establishment of the LPS-induced inflammation model in C57BL/6 mice, 2×10^6^ MC38 cells were injected through spleen. DNase 1 (100U/mouse) abrogation was then given daily. The mice were then sacrificed, and intrahepatic metastasis burden was assessed after 20 days. Experimental intrahepatic metastasis burden was assessed by calculating the percentage of hepatic tissue replaced by tumor (the hepatic replacement area, HRA).

### Mice model 3: *In vivo* evaluation of anti IL-8 on NETs-triggered metastasis capacity of CRC cells

2×10^6^ human HT29 CRC cells were treated with NETs in the presence of anti IL-8 (5μg/ml, R&D) or not. Cells were then injected into liver of null mice. Mice were then sacrificed and liver tumors were directly sized after 20 days.

### Isolation of neutrophils

Human neutrophils were isolated from the blood obtained from CRC patients and healthy donors by a widely used one-step gradient centrifugation method using Polymorph^Prep^ (Axis-Shield) according to instruction, and maintained in RPMI 1640 supplemented with 5% fetal calf serum (FCS) for immediate use. A purity of 90% was confirmed by flow cytometry using anti-CD15 antibody staining (BD Bioscience), with a viability rate over 95% by Trypan blue exclusion. Mice neutrophils were obtained from peripheral blood of C57/BL6 mice through density gradient centrifugation using Histopaque 1077/1119.

### *In vitro* assays on invasion, death rate, adhesion and proliferation of CRC cells

In the set of *in vitro* invasion assay, 1×10^5^ CRC cells in serum-free DMEM were seeded on the upper chamber of 8-μm Transwell system coated with Matrigel (BD) or not, and 5×10^5^ neutrophils/NETs with or without DNase 1 (100U/ml) were used. After 30 hours incubation, the contents of the upper chambers were aspirated, washed and cleared by a cotton swab. Cells on lower membranes were then stained with crystal violet. Cells invading through the membrane were quantified in 4 random fields. To study the cytotoxicity of NETs on CRC cell death, 1×10^5^ CRC cells were seeded on 24-well plates and incubated overnight. NETs with or without DNase 1 (100U/ml) were added to each well for 24 hours. Then cells were fixed and cell death rate was assessed using Tunel assay (Roche) according to the instruction.

For adhesion assay, 1×10^6^ human neutrophils were seeded on a 24-well plate and left intact or stimulated with 20 nM PMA with or without DNase 1 (100U/ml) for 4 hours to form NETs, then 1×10^5^ Dil-labeled CRC cells were added to each well. After 20 minutes incubation, each well was washed five times, followed by 4% PFA fixation. The adhered Dil-labeled cells were directly quantified under fluorescence microscopy from 5 random fields.

For proliferation assay, 2×10^3^ CRC cells were seeded on 96-well plates and incubated overnight. NETs with or without DNase 1 (100U/ml) were added to each well and incubated with CRC cells for 72 hours. The cell proliferation was measured every 24 hours using CCK8 kit (Beyotime) according to instruction. Anti-IL 8 (5μg/ml) was added 60 minutes prior to NETs treatment in selected experimental groups.

### Cell immunofluorescence staining

For NETs detection, 2×10^5^ neutrophils were seeded on poly-L-lysine-coated coverslips in 24-well plates to form NETs as described above, and then fixed with 4% PFA for 20 minutes. Subsequently, coverslips containing NETs were permeabilized with 0.1% Triton X-100 for 15 minutes at RT, washed with phosphate-buffered saline, and blocked with PBS containing 1% bovine serum albumin for 1 hour at RT. NETs were stained with primary antibody in blocking buffer at 4°C overnight. After wash, NETs were stained with matched fluorescence-conjugated secondary antibodies (Jackson; 1:600) in blocking buffer and finally stained with Hoechst33342 for nuclear(1:1000). Slides were then mounted with Fluoro-gel (Beyotime) and observed under fluorescence microscopy. Images were analyzed with ImageJ software. A similar procedure was performed for immunofluorescence staining of CRC cells. Primary antibodies used: H3cit (1:100, Abcam), MPO (1:100, Abcam), NF-κB (P65) (1:500, CST).

### Evaluation of *in vivo* adhesion of CRC cells in liver and lung

To evaluate *in vivo* adhesion of cancer cells, 5×10^5^ Dil-labeled MC38 cells were injected into the LPS-induced NETs models from spleen as described in establishment of LPS-induced NETs model. At indicated time point, livers were removed, embedded in OCT and prepared for frozen sections. The average number of adhered cancer cells was counted in at least 10 random high field images from 5 sections under fluorescent microscope.

### Tissue immunohistochemical and immunofluorescence staining

Immunohistochemical staining of paraffin-embedded sections was performed by the avidin-biotin-peroxidase complex method. Briefly, after rehydration and microwave antigen retrieval, primary antibodies were applied, incubated at 4°C overnight, and followed with secondary antibody incubation (GeneTech) at 37°C for 30 minutes. Staining was performed with 3, 30-diaminobenzidine tetra hydrochloride and counterstaining was performed with Mayer's hematoxylin. Immunofluorescence staining of NETs components in paraffin-embedded sections was performed similarly. Sections were proceeded with rehydration and antigen retrieval, followed by elimination of auto-fluorescence. Primary antibodies were then applied, incubated at 4°C overnight, and followed with fluorescence-conjugated secondary antibody incubation and Hoechst33342 stain of nuclear. Primary antibodies used: H3cit (1:100, Abcam), IL-8 (1:50, R&D). Photographs of 5 representative fields were captured and analyzed using software ImageJ with identical setting. NETs (marked as H3cit) and IL-8 were evaluated as percentage of area covered by positive staining.

### Quantitative real-time PCR

Total RNA was extracted using Trizol (Invitrogen), and reverse-transcribed into single-stranded cDNA using PrimeScript™ RT Reagent Kit (TaKaRa Biotechnology). Quantitative real time polymerase chain reaction (qRT-PCR) was performed with SYBR Green qPCR Master Mix (DBI Bioscience). Specific primers were provided in Supplementary Table. Expression levels were normalized against *β-actin* in each sample and then standardized as fold change. Primers used were listed in **Table S5**.

### Statistical analysis

The results are expressed as the means ± SEM. The statistical significance of differences between groups was determined by Student's t-tests. Pearson correlation test was used for correlation analysis. Kaplan-Meier method and Log-rank test were used for follow-up data. Graphpad statistical software (version 5.0) was used for all statistical analyses. All data were analyzed using two-tailed tests unless otherwise specified, and *P* < 0.05 was considered statistically significant.

## Results

### Neutrophils form increased extracellular traps (NETs) in CRC patients

**Figure 1:**
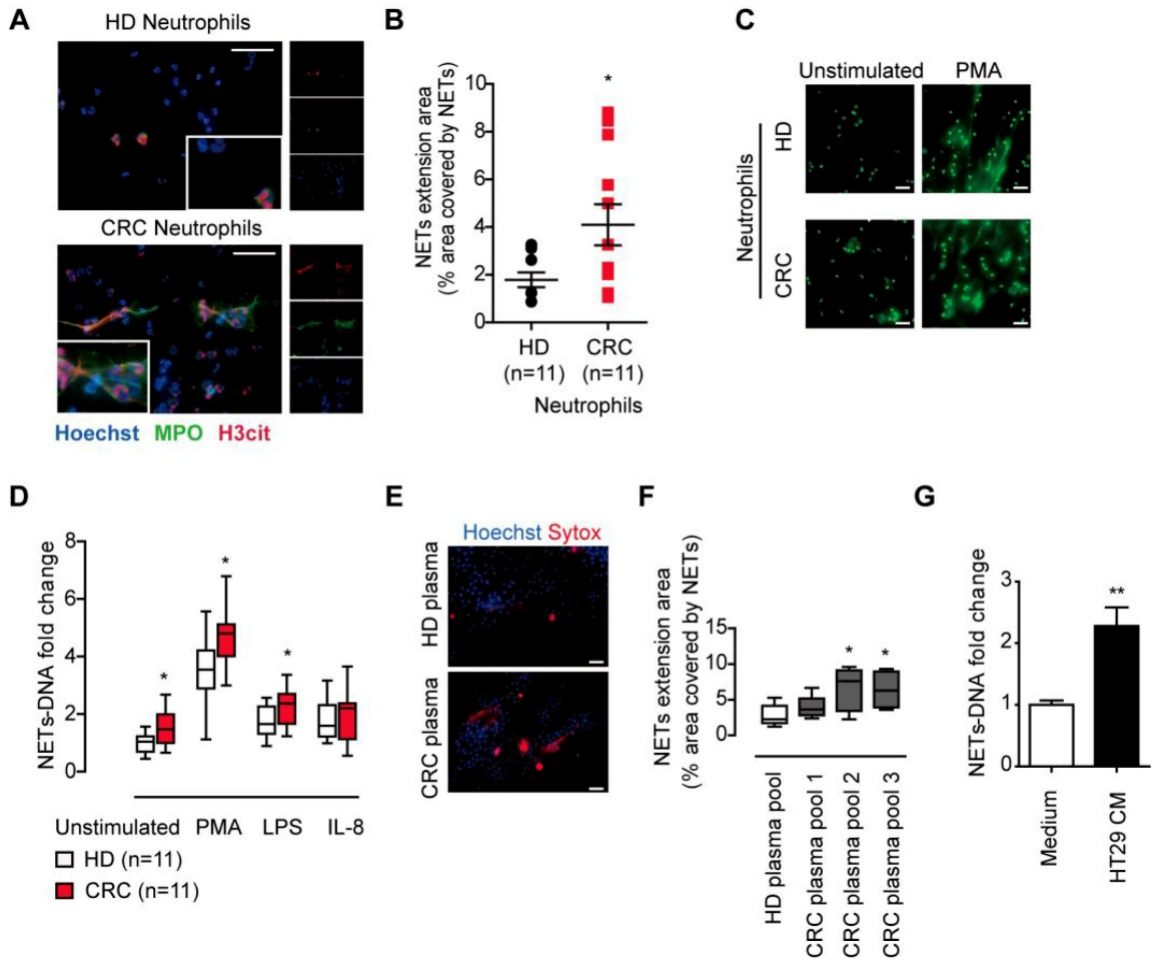
** CRC neutrophils presented enhanced NETs formation. (A)** Representative images of spontaneous NETs formation of neutrophils isolated from CRC patients but not HD. Neutrophils were incubated for 4h and NETs were stained for extracellular DNA (blue), MPO (green) and H3cit (red). Scare bar: 20μm. **(B)** NETs extension area analysis of neutrophils isolated from CRC patients and HD. **(C)** Representative images of NETs formation of CRC/HD neutrophils under indicated stimulus. Neutrophils were treated with stimulus for 4h and fixed, then NETs were stained with SytoxGreen for extracellular DNA. Scare bar: 20μm.** (D)** Quantification of NETs-DNA released from CRC/HD neutrophils under indicated stimulus. **(E)** Representative images of NETs formation from normal neutrophils in CRC plasma but not HD plasma. Scare bar: 50μm. **(F)** Quantification of NETs-DNA released form normal neutrophils in CRC plasma but not HD plasma. **(G)** Quantification of NETs-DNA released form normal neutrophils in HT29 CM. Data were presented as means ± SEM.

We first isolated fresh peripheral neutrophils and compare the NETs release from CRC patients and healthy donors. After incubation and observation under immunofluorescence, we found neutrophils from CRC patients underwent spontaneous NETs formation, while neutrophils from healthy donors formed fewer NETs **(Figure 1A)**. The NETs release from CRC patients' neutrophils was enhanced compared to that from healthy controls **(Figure 1B)**. Neutrophils under pathological conditions are often pre-stimulated, known as primed condition, and hyper-responsive to additional stimulus to form NETs. In line, NETs from CRC neutrophils were also higher than that from healthy controls under NETs stimulus PMA, LPS and IL-8 **(Figure 1C-D)**. These spontaneous NETs formation and hyper-responsiveness to NETs stimulus indicated some certain stimulus under CRC condition. We then incubated normal neutrophils in plasma from CRC patients and healthy controls, and found plasma from CRC patients favored NETs formation, indicating that certain soluble factor(s) from CRC cells released into circulation and stimulate neutrophils towards NETs formation **(Figure 1E-F)**. We further found that conditioned medium from human CRC cell line HT29 was also capable of inducing NETs from normal neutrophils, further supporting the presence of soluable NETs-inducing factor(s) from cancer cells** (Figure 1G)**. All together, these results showed that neutrophils formed enhanced NETs in CRC patients, and certain soluble factor(s) from CRC cells activated neutrophils towards NETs formation.

### NETs level correlates with CRC liver metastasis

**Figure 2:**
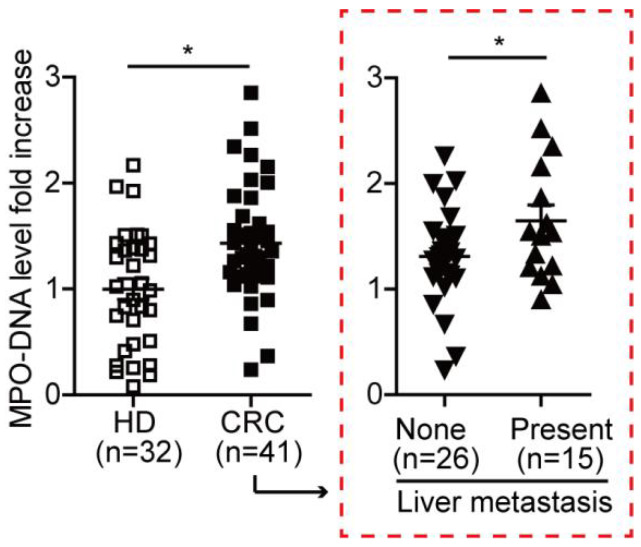
** MPO-DNA level was increased and correlated with CRC metastasis state.** MPO-DNA level was detected in serum samples from CRC (n=41) and HD (n=32) by Capture-ELISA. Data were presented as means ± SEM.

**Figure 3:**
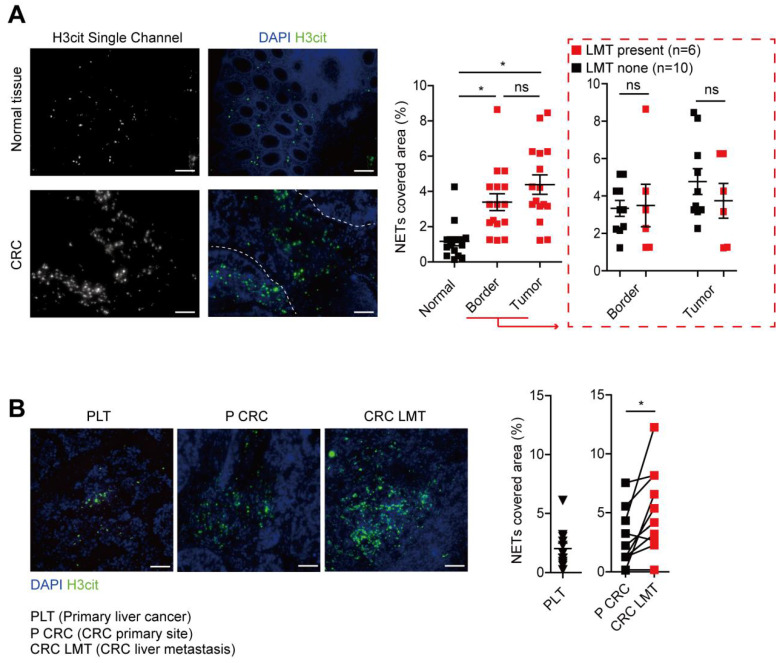
** NETs formed in CRC primary site and liver metastasis. (A)** Representative images and quantification NETs (H3cit) in CRC primary tumors and normal colons. Scare bar: 100μm.** (B)** Representative images and quantification of NETs (H3cit) in primary liver cancer and paired CRC primary tumors and liver metastasis. Scare bar: 100μm. Data were presented as means ± SEM.

To further study the NETs formation in CRC patients, we measured the MPO-DNA complex (NETs product) by capture-ELISA in sera samples from CRC patients and healthy controls. An elevated MPO-DNA level was observed in CRC sera, indicating part NETs formation already happened in circulation. To be noticed, we further found that among CRC patients, those with liver metastasis displayed a further elevation in serum MPO-DNA level compared to those with no liver metastasis **(Figure 2)**. We further sought pathological evidence of NETs in CRC tissue samples. We found NETs mainly distributed around tumor border and within tumor in CRC primary site, where the normal colon displayed few NETs distribution **(Figure 3A)**. To be noticed, there was no significant difference of NETs distribution among the primary site of CRC with or without liver metastasis, around tumor border or within tumor. But there were significantly more NETs in paired CRC liver metastasis site than the primary tumor site in the same patients having simultaneous resection. As another proof, relative similar level of NETs was found between primary liver cancer (hepatocellular carcinoma) and CRC primary tumor, but when comparing NETs in different liver cancers, more NETs were found in CRC liver metastasis than primary liver cancer **(Figure 3B)**. These indicated that although NETs are released from recruited neutrophils as primary CRC tumors progress, NETs formation in pre-metastasis liver are more important for the onset of liver metastasis rather than NETs in primary tumor. Collectively, these results showed NETs correlate with CRC liver metastasis and may be a key metastatic initiation factor.

### Increased NETs fuel CRC liver metastasis

**Figure 4:**
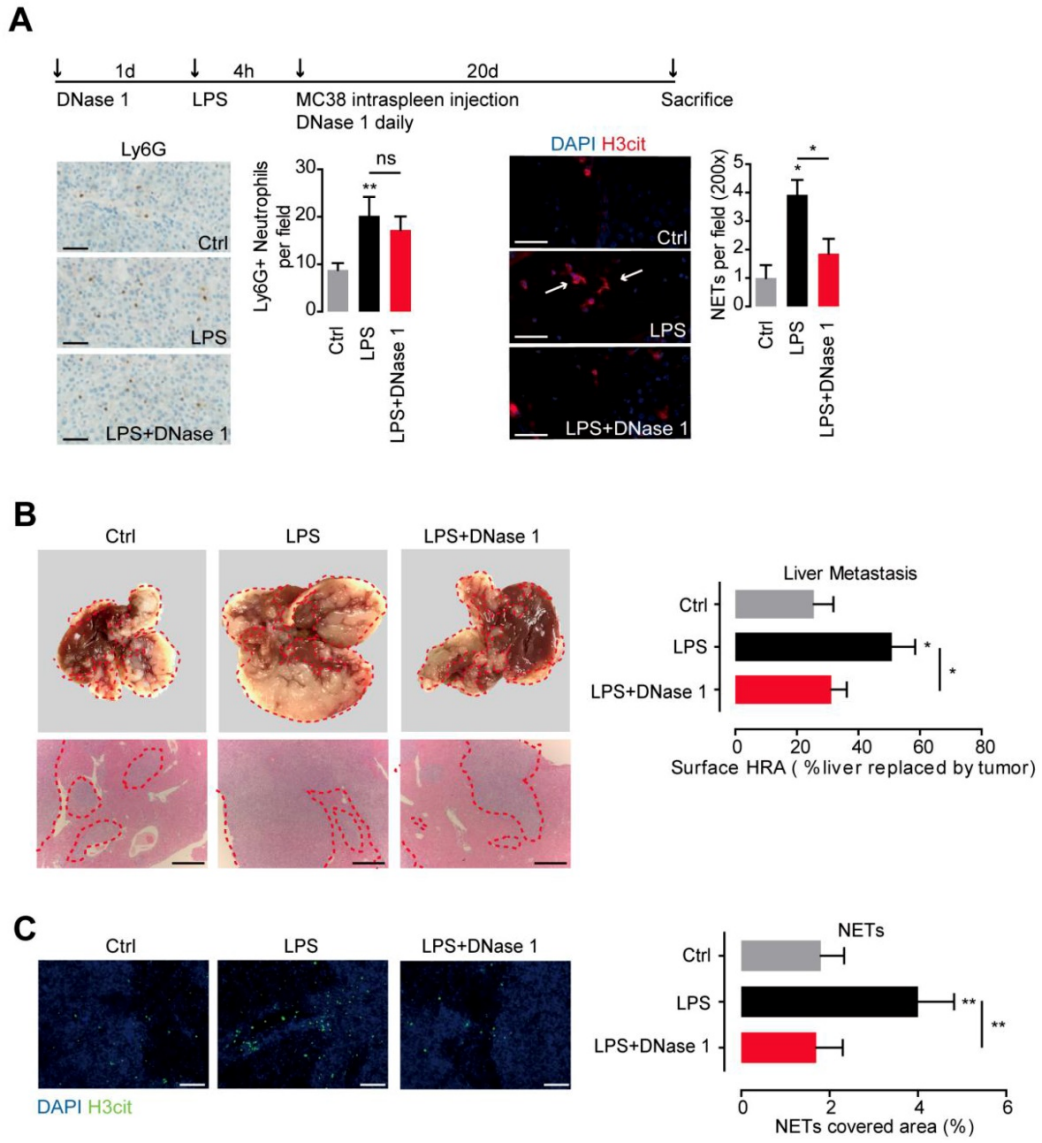
** NETs fueled CRC metastasis in mice. (A)** LPS-induced NETs model through peritoneal LPS administration in C57BL/6 mice (upper panel). LPS mobilized neutrophils and induced NETs in liver. DNase 1 abrogated NETs but not neutrophils recruitment. Representative immunofluorescence images and quantification of neutrophils (Ly6G, left panel) and NETs (H3it, right panel) in liver were shown. Symbol → indicated typical NETs-forming neutrophils. Scare bar: 50μm.** (B)** CRC experimental metastasis was increased in the LPS-induced NETs model and abrogated by DNase 1. Representative images and quantification of gross CRC liver metastasis by intraspleen injection of mice MC38 cells. Scare bar: 200μm.** (C)** Representative images and quantification of NETs (as H3cit) *in situ*. Scare bar: 100μm. Data were presented as means ± SEM.

To further illustrate the role of NETs in CRC metastasis, we conducted the experimental liver metastasis in a well adopted LPS-induced NETs model. Systemic administration of LPS effectively recruited neutrophils and activated NETs formation in mice liver. DNase 1, by effectively wrecking NETs, did not affect neutrophils recruitment, proving the well establishment of the LPS-induced NETs model **(Figure 4A)**. Moreover, we found in the presence of NETs, significant more liver metastasis formed after loading of mice MC38 CRC cells through spleen. In line, more NETs formed within the established metastasis tumor in the LPS-induced NETs model **(Figure 4B)**. DNase 1 by wrecking formed NETs, effectively abolished the increased liver metastasis **(Figure 4B)**. These results showed the increased NETs fuel CRC liver metastasis and NETs releasing may be an important pattern of neutrophils to promote CRC metastasis.

### NETs trap disseminated CRC cells to optimize their seeding in liver

**Figure 5:**
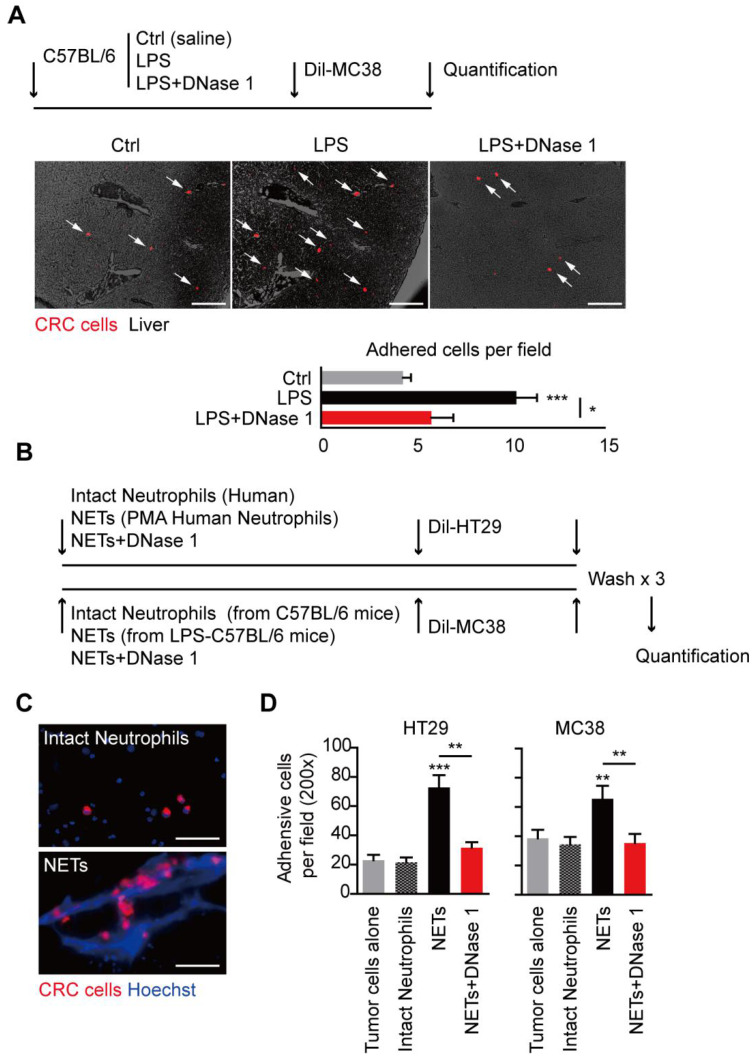
** Trapping role of NETs on CRC cells both *in vivo* and *in vitro*. (A)** Representative images and quantification of early adhesion of Dil-CRC cells *in vivo*. Scare bar: 200μm. **(B)** Systemic illustration of assaying trapping of NETs on CRC cells *in vitro*. **(C)** Representative images of NETs trapping HT29 cells. Scare bar: 50μm. **(D)** Quantification of adhered CRC cells on NETs compared and intact neutrophils. Data were presented as means ± SEM.

NETs were originally discovered as innate immune defense to trap and restrict invading pathogens with their widely extending web-like structure. The web-like structure may also serve as an adhesion platform for host and cancer cells. We observed that in LPS-induced NETs model, significantly more MC38 CRC cells adhered in hepatic sinus after spleen adoption in the presence of NETs, and this trend of increased early CRC cells seeding was consistent of the increased final establishment of liver metastasis **(Figure 5A)**. In line with the result in gross metastasis observation, this trend of increased early adhesion was abolished with DNase 1 adoption *in vivo* **(Figure 5A)**. We further illustrated the trapping role of NETs on CRC cells *in vitro* **(Figure 5B)**. In an *in vitro* adhesion system, we observed that CRC cells trapped in the large web-like structure of NETs, while there were only few physical interactions between CRC cells and intact neutrophils forming no NETs **(Figure 5C)**. More CRC cells were adhered on NETs than intact neutrophils monolayer, while intact neutrophils increased no adhesion rate of HT29 and MC38 CRC cells **(Figure 5D)**. Again, the increased trend could also be abolished by DNase 1 *in vitro* **(Figure 5D)**. These result showed the trapping role of NETs on CRC cells may be an initiating and essential step for fueling liver metastasis.

### NETs promote the metastatic behavior of trapped CRC cells

**Figure 6:**
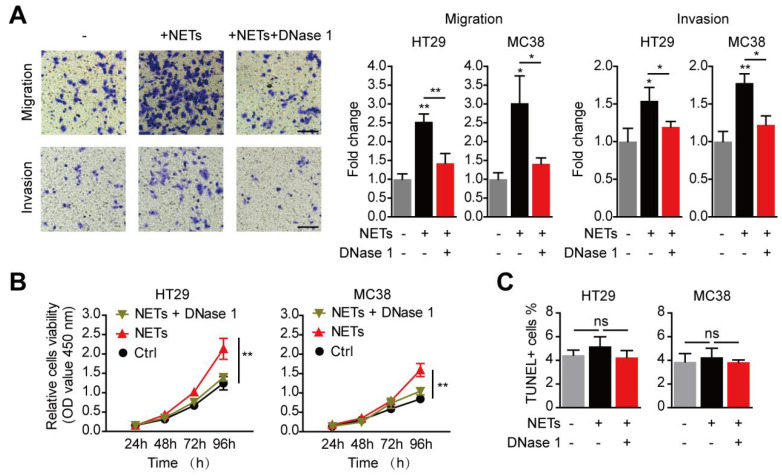
** Enhanced metastasis capacity of trapped CRC cells by NETs. (A)** NETs promoted migration and invasion of CRC cells in the Transwell system, and this was abrogated by DNase 1. Representative images (HT29) and quantification were shown. Scare bar: 50μm. **(B)** NETs promoted proliferation of CRC cells in the CCK8 assay, and this was abrogated by DNase 1.** (C)** NETs hardly exerted potential cytotoxicity of NETs on trapped CRC cells. Data were presented as means ± SEM.

We then isolated NETs from stimulated neutrophils to study the impact of NETs on trapped CRC cells. Consistent with the reports of invasion-promotion capacity of NETs on some cancer cells^20^,^28^, we found HT29 and MC38 CRC cells migration and invasion capacity were raised by NETs adoption* in vitro* **(Figure 6A)**. Moreover, *in vitro* proliferation of CRC cells was also significantly raised by NETs** (Figure 6B)**. When NETs were wrecked by DNase 1, there was no increase in migration, invasion or proliferation capacity of CRC cells **(Figure 6A-B)**. Given that previous reports have revealed certain cytotoxicity of NETs on endothelial cells and other host cells^29^, we next studied whether such potential anti-tumor effects may happened on CRC cells trapped by NETs. In fact, neither NETs nor DNase 1 exerted any alteration on cell viability of CRC cells *in vitro* **(Figure 6C)**. These results showed that NETs trapped disseminated CRC cells, altered no viability after their seeding, but increased the invasion and proliferation capacity of trapped CRC cells.

### Over-production of tumorous IL-8 mediate the increased CRC metastatic capacity caused by NETs

**Figure 7:**
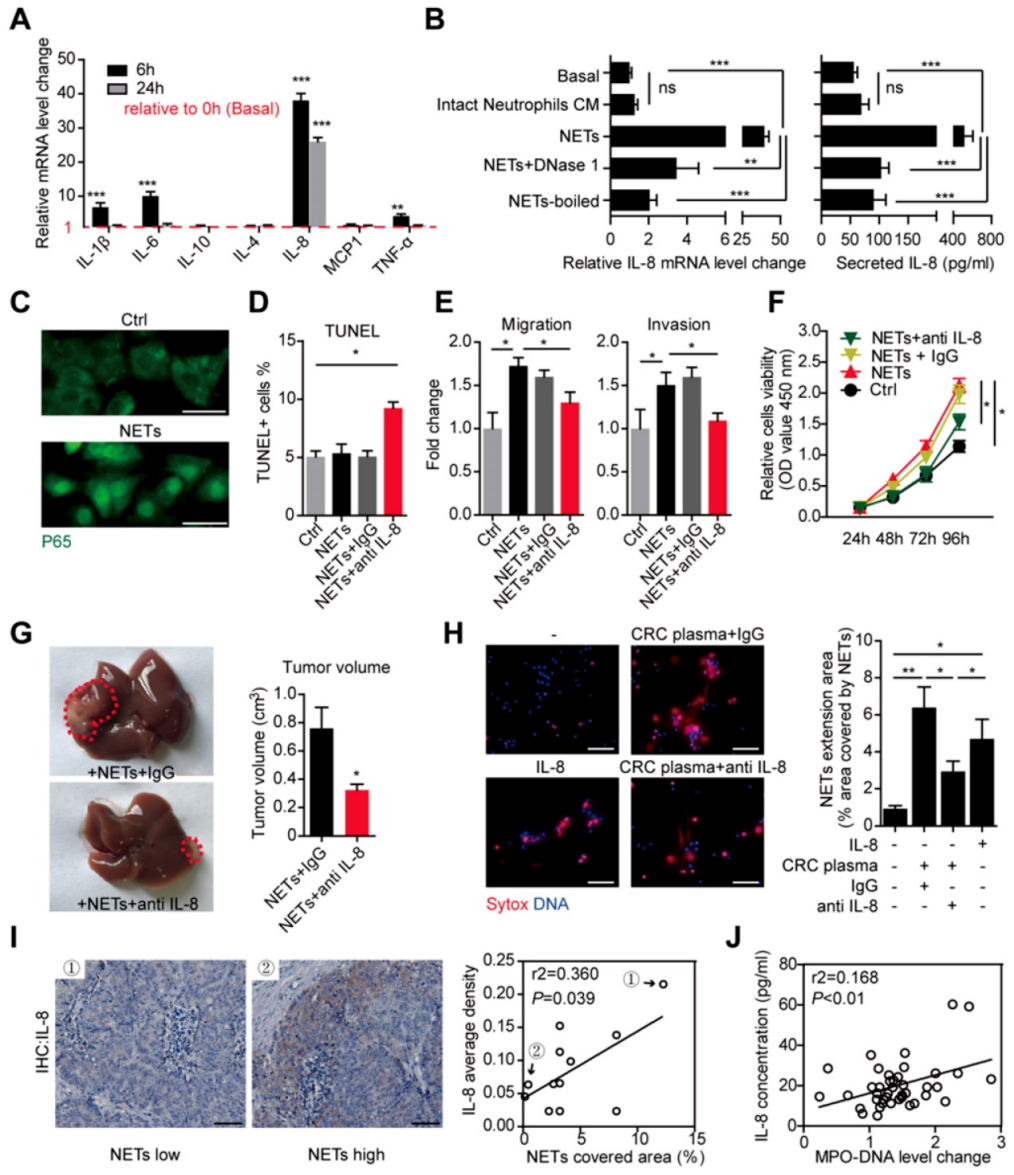
** IL-8 mediated both the NETs-triggered metastasis capacity and cancer-enhanced NETs formation in CRC. (A)** Detection of several cytokine mRNA level in HT29 cells treated with NETs by RT-PCR.** (B)** NETs, but not intact neutrophils CM, elevated the mRNA and secretion level of IL-8, and this was abrogated by DNase 1 or total boil of NETs.** (C)** Immune-staining revealing trans-location of P65 into nuclear in NETs-treated HT29 cells. Scare bar: 20μm. **(D)** NETs caused CRC cells death in the presence of anti IL-8 by TUNEL assay.** (E)** Anti IL-8 abrogated the NETs-enhanced migration and invasion of CRC cells. **(F)** Anti IL-8 abrogated the NETs-enhanced proliferation of CRC cells. **(G)** Anti-IL8 diminished the tumors formed in liver after NETs treatment. HT29 cells pretreated with NETs plus anti-IL8/IgG were injected into liver and grew for 2 weeks. Representative images of liver tumors were shown. **(H)** IL-8 stimulated NETs formation in normal neutrophils, and CRC plasma-induced NETs were partly reduced by anti IL-8. Normal neutorphils were isolated and incubated as indicated for 4h in cell-permeable dye Hoechst and cell impermeable dye Sytox for visualization. Represntative images of NETs and quantification were shown. Scare bar: 50μm. **(I)** Representative IL-8 immunohistochemistry images and Pearson correlation between IL-8 and NETs expression in CRC liver metastasis. Scare bar: 50μm. **(J)** Pearson correlation between serum IL-8 and MPO-DNA level in CRC patients. Data were presented as means ± SEM

We further studied the underlying mechanism of how NETs promote the metastatic capacity after trapping disseminated CRC cells. Enlightened by the reports that extracellular DNA has certain inflammatory-stimulation role on macrophage 30, we assumed certain inflammatory response might mediate the increased metastatic capacity caused by NETs. We performed RT-PCR on several inflammatory mediators production of HT29 cells treated by NETs. We found that mRNA level of cytokines such as IL-1β, IL-6, IL-8 and TNF-α displayed certain elevation after NETs treatment in HT29 cells, among which the elevation of IL-8 appeared more significant and consistent **(Figure 7A)**. We then focused on IL-8 and confirmed the tumorous IL-8 elevation by NETs in both the mRNA level and cytokine production level **(Figure 7B)**. However, this elevation of IL-8 was not seen in HT29 cells incubated with conditioned medium (CM) from intact neutrophils forming no NETs **(Figure 7B)**. Abrogating NETs by DNase 1 or heat-boil effectively abolished the tumorous IL-8 elevation **(Figure 7B)**. IL-8 is a key cytokine in inflammatory regulation and displays some tumor-promoting roles. We observed that NETs caused trans-location of P65 (NF-κB pathway) into nuclear **(Figure 7C)**, which marked an activation of inflammation response as well as potential biological triggering of tumorous metastatic activity. We then asked whether IL-8 accounts for the increased metastatic capacity triggered by NETs. We found adoption of neutralizing antibody against IL-8 extruded the potential cytotoxicity of NETs on trapped cells, while NETs alone without interfering IL-8 had no such cytotoxicity **(Figure 7D)**. Moreover, neutralizing antibody against IL-8 also abrogated the increased invasion and proliferation capacity of NETs-treated HT29 cells **(Figure 7E-F)**. We further asked whether interfering IL-8 might abolish the NETs-raised metastatic capacity* in vivo*. We injected NETs-treated HT29 cells with or without IL-8 neutralizing antibodies into mice liver. Consistent with the* in vitro* results, the NETs-treated HT29 cells with IL-8 interfered formed less tumor **(Figure 7G)**, which further supported the role of tumorous IL-8 overproduction in the increased metastatic capacity caused by NETs.

### Tumorous IL-8 mediate the increased NETs formation in CRC patients

**Figure 8:**
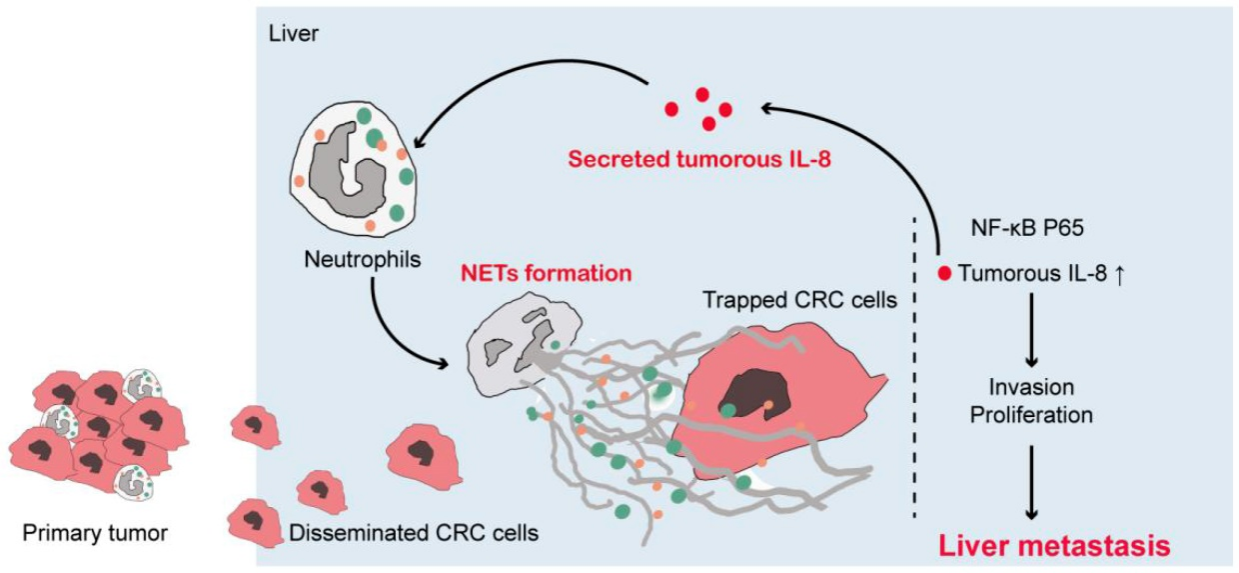
Illustration of IL-8 mediating a positive loop connecting increased NETs and CRC liver metastasis

IL-8 can activate neutrophils and promote NETs formation 31. Given that NETs triggered tumorous IL-8 production, we assumed that the increased IL-8 might explain the increased NETs formed in CRC neutrophils in a positive feedback manner. We used pooled CRC plasma and IL-8 to induce NETs formation. As we assumed, CRC plasma and IL-8 both effectively triggered NETs from normal neutrophils, as demonstrated as extruded DNA mash by immunofluorescence and quantification **(Figure 7H)**. Adoption of IL-8 neutralization partly attenuated NETs formation induced by CRC plasma **(Figure 7H)**. To be noticed, the fact that IL-8 neutralization failed to completely abolished CRC plasma-induced NETs formation revealed some other tumorous soluable factors in addition to IL-8 also responsible for the increased NETs in CRC patients. Furthermore, we found a close correlation between NETs and IL-8 expression in CRC liver metastasis loci **(Figure 7I)**. Consistently, sera MPO-DNA and IL-8 level were also in correlation **(Figure 7J)**. Collectively, these supported a positive loop connecting NETs to tumorous IL-8 to fuel CRC liver metastasis **(Figure 8)**.

## Discussion

CRC metastasis is closely related to innate and adaptive immune response. Neutrophils are major component of innate immune system and actively participate in malignant disorders 4,32,33. Both pro- and anti-tumor capacity of neutrophils have been discovered, depending on different cancer types and disease progression 32. NETs have been implied by separated studies to participate in progression of certain types of solid tumor including CRC. However, solid clinical evidence of NETs on CRC metastasis based on clinical samples is less involved. Moreover, how CRC activates neutrophils to form NETs and how NETs interact with CRC metastatic capacity are still to be illustrated. Thus in this study, by various detection means on different clinical samples, we provided solid evidences that neutrophils from CRC patients presented enhanced capacity to form NETs. Moreover, we found that CRC derived IL-8 activated NETs formation, which optimized the seeding of disseminated CRC cells from primary site into pre-established metastasis site, and further boosted tumor invasion and proliferation. More importantly, we further discovered that elevated tumorous IL-8 in response to NETs was the key to the boosted metastatic behaviour of CRC cells, and IL-8 in turn stimulated neutrophils to form NETs, thus forming a metastasis-favourable positive loop between neutrophils and CRC cells.

Cancer cells often hijacked host cells to create a metastatic-favourable macro/micro-environment 34. However, the role of neutrophils in cancer metastasis is still controversial across different studies. Some suggested that mobilized neutrophils as rapid response in host defense system inhibit through releasing cytotoxic substance 7. Others proposed that neutrophils shift into a metastasis-favorable subtype as tumor progress 35,36. Despite a increasing recognition of neutrophils in CRC progression, how neutrophils interact CRC, especially CRC with liver metastasis, is less recognized. NETs are essential and unique function of neutrophils, and actively participate in several pathological disorders including malignancy. In CRC, separated reports revealed a possible involvement of NETs in CRC progression. In this study we aimed to further elucidate the relationship between NETs and CRC liver metastasis. We first focused on the NETs-forming capacity between neutrophils from CRC patients and normal ones. The results showed that freshly isolated CRC neutrophils have enhanced NETs-forming capacity in comparison to healthy controls, and this was concluded from the spontaneous NETs formation and over-production of NETs with stimulus. These results were in line with a previous study reporting an enhanced NETs formation in CRC 18,37. But assessment of NETs in CRC based on metastasis state is not yet involved. We first assessed serum MPO-DNA complex level to quantify NETs in CRC patients, and found MPO-DNA level was consistently elevated in CRC patients when compared to healthy controls. To further support a role of NETs in CRC metastasis, we specifically divided CRC patients according to the presence of liver metastasis or not to specifically, and confirmed that the MPO-DNA level was further increased with the onset of liver metastasis. In one report using tissue immune-staining of NETs, it was revealed that recruited neutrophils in CRC primary tumor underwent NETs formation 38. In line, we proved more NETs were found in CRC primary site but not in normal colon. We then assessed whether there was a discrepancy of NETs in CRC with or without liver metastasis. Interestingly, no significant difference of NETs was observed in primary site of CRC with or without liver metastasis. We thus proposed that rather than in primary site, NETs are more important in liver to affect metastasis, as we observed large amount of NETs in CRC liver metastasis site. However, it was ethically unavailable to compare amount of formed NETs between CRC liver metastasis and normal liver of CRC patients with no metastasis. We then made an alternative comparison between CRC liver metastasis and primary liver cancer (hepatocellular carcinoma), and found a higher amount of NETs in metastatic liver tumors than primary one. Our findings suggested that NETs were closely relevant to onset of liver metastasis in CRC patients, and NETs might play an more essential role in pre-metastatic liver rather than primary site to help metastasis.

Consistent with a previous study suggesting that hepatic ischemia-reperfusion injury caused NTEs to favour cancer metastasis, here we used a well-established LPS-induced NETs mice model to elucidate the effect of NETs on CRC liver metastasis. Correlated with previous reports, administration of LPS rapid mobilize neutrophils and NETs in liver. NETs in liver caused more liver metastasis after loading of CRC cells. A detail analysis of NETs in metastasis cascade by both *in vitro* and *in vivo* means suggested that NETs trapped CRC cells, thus offering CRC cells the better chance to seed in the liver. Some studies showed unregulated NETs have cytotoxicity on endothelial cells and cause organ dysfunction. The actual role of NETs on trapped CRC cells remained unclear, and both pro- and anti-tumor effect of NETs on CRC cells have been raised 38. Here we observed no such cytotoxicity of NETs on CRC cells, eliminating a possibility that NETs trap and kill CRC cells like invading pathogen. On the contrary, CRC cells trapped by NETs presented higher proliferation and invasion capacity, which further optimize the formation of liver metastasis after CRC cells seeding in liver.

How NETs are provoked in CRC patients and how NETs promote CRC metastatic capacity are largely unknown. NETs are strong inflammatory stimulus, and can license the production of cytokine in host cells such as macrophages 30,41,42. In light of this, we observed a similar elevation of several inflammatory mediators in CRC cells treated with NETs. IL-8 is a classical multifunctional inflammatory mediator on both host and cancer, and several groups has demonstrated high level of circulating IL-8 in cancer patients 24. Moreover, in CRC and other tumor cells, overproduced IL-8 marked a more malignant phenotype with worse outcome 25. IL-8 favors metastatic spread, invasion, tumor growth and angiogenesis 39,40. As supportive evidence, it is found extracellular chromatin released by pancreatic cancer cells can elevate metastasis-favourable IL-8 secretion 43,44. Moreover, when blocking IL-8, CRC cells failed to gain any enhanced proliferation nor invasion capacity from NETs. These data uncovered that IL-8 is the key factor in NETs-aroused CRC malignant behaviour.

Moreover, IL-8 induced migration of neutrophils in inflammation and cancer environment. A tumorous IL-8 overproduction also expands granulocytic myeloid-derived suppressor cells (Gr-MDSC) in mice and patients. In addition, IL-8 served as a potent NETs inducer in lymphoma and malignant cachexia. We thus assumed IL-8 provoked NETs in CRC. Our present study showed NETs stimulated tumorous IL-8 production in CRC cells, and the overproduced tumorous IL-8 in turn activated neutrophils and enhanced NETs formation, which formed a positive feedback connecting NETs and CRC cells to optimize liver metastasis. Consistently, this correlation between IL-8 and NETs was further verified in clinical samples of CRC liver metastasis.

Taken together, in this study we provided solid evidences that CRC neutrophils underwent pronounced NETs formation, and NETs correlated with CRC liver metastasis. NETs favored CRC liver metastasis. More importantly, we also uncovered a novel mechanism that NETs trapped CRC cells, and increased metastatic behavior of tumor cells through triggering tumorous IL-8 elevation, and elevated IL-8 in turn recruited more neutrophils to form enhanced NETs, which formed a positive loop connecting CRC and NETs-forming neutrophils to promote CRC liver metastasis **(Figure 8)**. Our findings added to the present knowledge of how neutrophils intact with CRC cells, especially in liver metastasis, and also reveal a novel mechanism of metastasis-supporting NETs on CRC cells through IL-8 as an appealing therapeutic strategy against CRC liver metastasis by targeting NETs and IL-8.

## Supplementary Material

Supplementary tables.
