# Supplementary material for: IL-8 mediates a positive loop connecting increased neutrophil extracellular traps (NETs) and colorectal cancer liver metastasis
Source: J Cancer. 2020 May 18;11(15):4384–96. doi: 10.7150/jca.44215 (PMC7255375; doi:10.7150/jca.44215)
Supplement: Supplementary file 1 — Supplementary tables. [file jcav11p4384s1.pdf]

Table S1. Clinical characteristics of patients for NETs analysis of primary tumour site (n=16)

Characteristics	N(%)
Average age	61.5
Male/female	11/5
Primary site	
left-sided colon	7(44)
right-sided colon	8(50)
rectum	1(6)
Tumor stage	
T1	2(13)
T2	5(31)
T3	4(25)
T4	5(31)
lymph node status	
N0	3(19)
N1	7(44)
N2	5(31)
Liver metastasis	
None	10(63)
Yes	6(38)

Table S2. Clinical characteristics of patients for sera MPO-DNA detection (n=41)

Characteristics	N(%)
Average age	66.2
Male/female	29/12
Primary site	
left-sided colon	21(51)
right-sided colon	14(34)
rectum	6(15)
Liver metastasis	
None	26(63)
Yes	15(37)

Table S3. Clinical characteristics of patients for NETs detection in paired primary site and liver metastasis(n=10)

	Age	Gender	Primary tumor			Liver metastasis	
			Location[a]	T stage	N status	Lesion>3	Largest lesion>5cm
Pt1	65	M	1	T3	N1	y	n
Pt2	53	M	1	T3	N1	y	n
Pt3	59	F	2	T4	N2	n	n
Pt4	67	M	1	T2	N1	n	n
Pt5	61	F	2	T4	N1	y	y
Pt6	72	M	3	T4	N2	y	y
Pt7	49	M	2	T3	N1	n	n
Pt8	55	M	1	T2	N1	n	n
Pt9	58	F	3	T4	N2	n	y
Pt10	60	M	2	T4	N2	n	y

a:1:left-sided, 2:right-sided 3:rectum

y: yes n: no

Table S4. Clinical characteristics of patients for NETs analysis of primary liver cancer (HCC, n=10)

**Characteristics**	**N(%)**
Average age	65.5
Male/female	7/3
HBsAg	
Negative	4/40
Positive	6/60
Liver cirrhosis	
No	5/50
Yes	5/50
Tumor size	
≤ 5cm	7/70
＞ 5cm	3/30
Tumor number	
Single	9/90
Multiple	1/10
Tumor encapsulation	
None	7/70
Complete	3/30
Vascular invasion	
No	6/60
Yes	4/40

Table S5. Real-time PCR primers used in the study

Primers	Seqeunces (5’-3’)
IL-8	F-CTCCAGCCACACTCCAACAGA
	R-CACCCTAACACAAAACACGAT
IL-6	F-CCACGGCCTTCCCTACTTC
	R-CTGTTGGGAGTGGTATCCTCTGT
TNF-α	F-GACGTGGAACTGGCAGAAGAG
	R-TCGGACAAGCAGGAATGAGA
IL-10	F-AGCCTTGCAGAAAAGAGAGC
	R-TGGAGTCCAGCAGACTCAAT
IL-1β	F-CTAAAGTATGGGCTGGACTG
	R-AGCTTCAATGAAAGACCTCA
IL-4	F-AAGCAAAAAGCCAGCAGCAGCC
	R-ACAAAGTTTCAGCATAGGAAATTAC
MCP-1	F-TAGGGAAGTTCCACAAAGTTAAAAAC
	R-GCCACACCAGCACACAGAAG
